# Supplementary material for: Mortality and Predictive Factors for Death Following the Diagnosis of Interstitial Lung Disease in Patients with Rheumatoid Arthritis: A Retrospective, Long-Term Follow-Up Study
Source: J Clin Med. 2025 Feb 19;14(4):1380. doi: 10.3390/jcm14041380 (PMC11855988; doi:10.3390/jcm14041380)
Supplement: Supplementary file 1 [file jcm-14-01380-s001.zip › Supplementary Table S1.pdf]

Table S1. DMARD use during follow-up in overall RA patients

	With RA-ILD (n = 78)	Without RA-ILD (n = 703)	*p**
DMARD use, number (%)			
MTX	46 (59.0)	640 (91.0)	<0.001
Other csDMARDs[†]	46 (59.0)	228 (32.4)	<0.001
bDMARDs			
TNF inhibitors	41 (52.6)	346 (49.2)	0.63
IL-6 inhibitors	41 (52.6)	225 (32.0)	<0.001
Abatacept	27 (34.6)	115 (16.4)	<0.001
JAK inhibitors[‡]	28 (35.9)	273 (38.3)	0.71
Length of exposure, years, mean (SD)			
MTX	5.7 (5.0)	8.3 (6.9)	0.013
Other csDMARDs[†]	4.3 (4.3)	4.7 (3.9)	0.56
bDMARDs			
TNF inhibitors	3.4 (4.0)	4.0 (4.5)	0.43
IL-6 inhibitors	2.4 (3.1)	3.3 (3.4)	0.15
Abatacept	2.0 (2.5)	2.7 ‘3.0)	0.28
JAK inhibitors[‡]	2.4 (2.6)	3.5 (2.9)	0.053

[†]Included tacrolimus, leflunomide, sulfasalazine, and bucillamine.

[‡]Included tofacitinib, peficitinib, baricitinib, upadacitinib, and filgotinib.

RA, rheumatoid arthritis; ILD, interstitial lung disease; RA-ILD, RA-associated ILD; MTX, methotrexate; DMARDs, disease-modifying anti-rheumatic drugs; csDMARDs, conventional DMARDs; bDMARDs, biological DMARDs; JAK, Janus kinase; TNF, tumor necrosis factor; IL-6, interleukin-6; SD, standard deviation
