# Supplementary material for: Mortality and Predictive Factors for Death Following the Diagnosis of Interstitial Lung Disease in Patients with Rheumatoid Arthritis: A Retrospective, Long-Term Follow-Up Study
Source: J Clin Med. 2025 Feb 19;14(4):1380. doi: 10.3390/jcm14041380 (PMC11855988; doi:10.3390/jcm14041380)
Supplement: Supplementary file 1 [file jcm-14-01380-s001.zip › Supplementary Table S2.pdf]

Table S2. DMARD use during follow-up in RA-ILD patients

	With MTX use (n = 46)	Without MTX use (n = 32)	*p**
DMARD use, number (%)			
Other csDMARDs†	22 (47.8)	24 (75)	0.020
bDMARDs			
TNF inhibitors	32 (69.6)	9 (28.1)	<0.001
IL-6 inhibitors	23 (50)	18 (56.3)	0.59
Abatacept	12 (26.1)	15 (46.9)	0.060
JAK inhibitors‡	16 (34.8)	12 (37.5)	0.81
Length of exposure, years, mean (SD)			
Other csDMARDs†	3.1 (2.8)	5.4 (5.1)	0.070
bDMARDs			
TNF inhibitors	3.5 (4.1)	3.1 (3.7)	0.76
IL-6 inhibitors	1.7 (2.9)	3.4 (3.2)	0.080
Abatacept	2.4 (2.2)	1.7 (2.7)	0.49
JAK inhibitors‡	2.3 (2.8)	2.5 (2.2)	0.84

†Included tacrolimus, leflunomide, sulfasalazine, and bucillamine.

‡Included tofacitinib, peficitinib, baricitinib, upadacitinib, and filgotinib.

RA, rheumatoid arthritis; ILD, interstitial lung disease; RA-ILD, RA-associated ILD; MTX, methotrexate; DMARDs, disease-modifying anti-rheumatic drugs; csDMARDs, conventional DMARDs; bDMARDs, biological DMARDs; JAK, Janus kinase; TNF, tumor necrosis factor; IL-6, interleukin-6; SD, standard deviation
