# Supplementary figures and images for: Mortality and Predictive Factors for Death Following the Diagnosis of Interstitial Lung Disease in Patients with Rheumatoid Arthritis: A Retrospective, Long-Term Follow-Up Study
Source: J Clin Med. 2025 Feb 19;14(4):1380. doi: 10.3390/jcm14041380 (PMC11855988; doi:10.3390/jcm14041380)

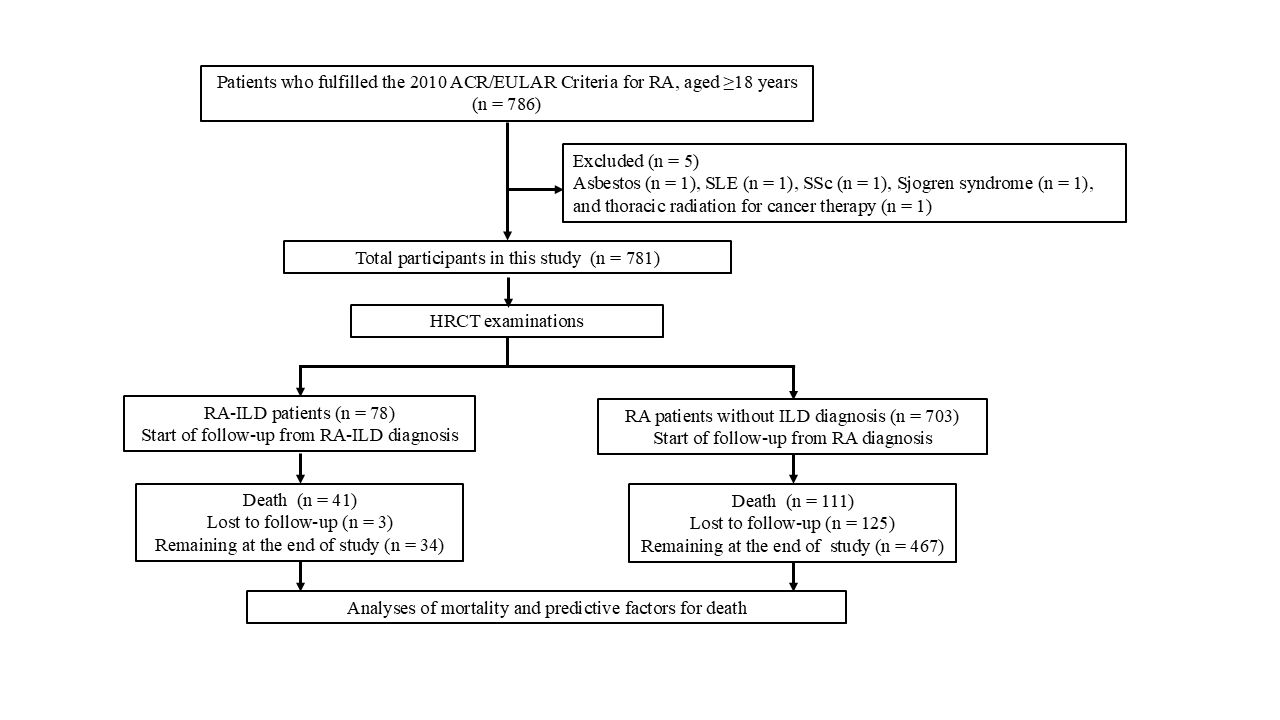

Supplement: Supplementary file 1 [file jcm-14-01380-s001.zip › Supplementary Figure S1.tif]

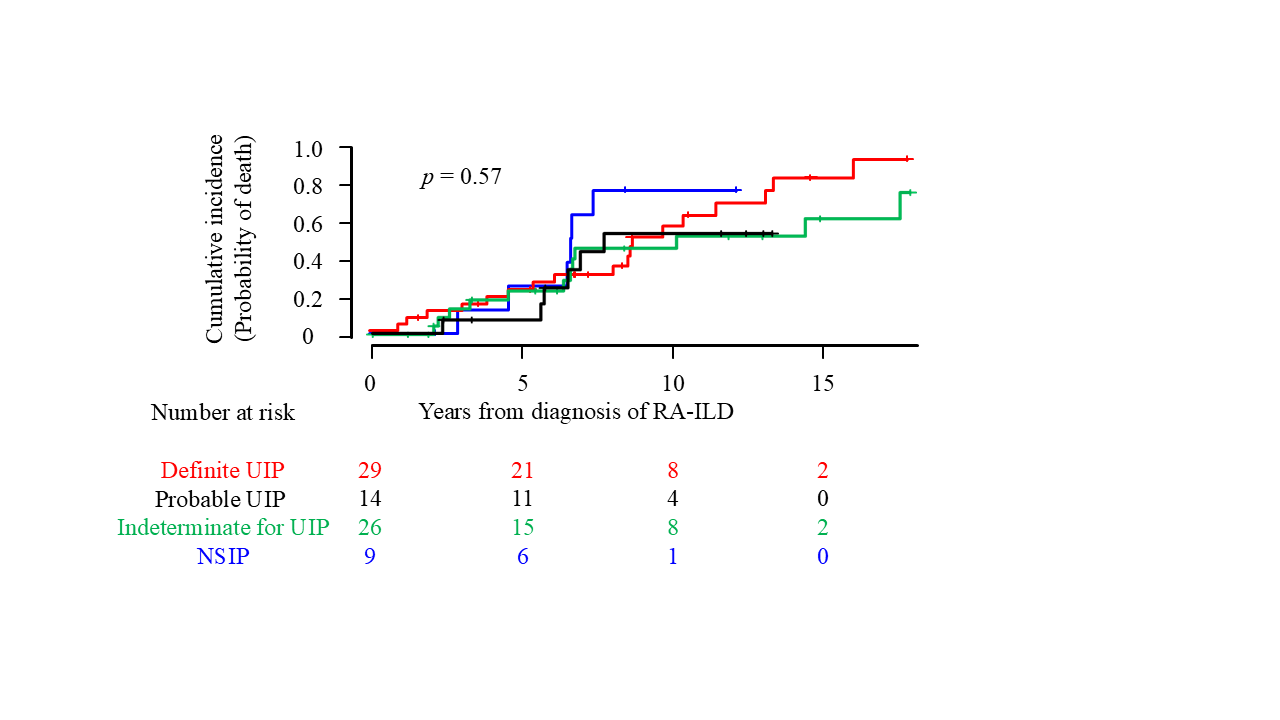

Supplement: Supplementary file 1 [file jcm-14-01380-s001.zip › Supplementary Figure S2.tif]
